# R. L. Polk & Co.’s Dental Register of the United States

**Published:** 1893-11

**Authors:** 


### Bibliography.

R. L. Polk & Co.'s Dental Register of the United States. Comprising List of Dentists arranged by States, giving Post-Office Address, etc.; also list of Dentists in the United States, arranged alphabetically. Vol. I. Published by R. L. Polk & Co., Detroit, Baltimore, Chicago, 1893.

The dental profession, and especially those who needed a good directory of dentists, can be congratulated that at last one is presented worthy of confidence. When the publishers of this work announced their intention of devoting all their resources and extended experience in its production, it was the universal expectation that they would accomplish all they promised to perform. That these promises have been fulfilled is amply demonstrated in the book given to the profession.

It comprises a volume of 704 pages, and, it is needless to say, filled with important information from the first page to the last.

The labor of compiling such a book can hardly be appreciated by those entirely unfamiliar with the methods required to secure information in this direction. It was undertaken and carried to completion with fewer errors than could have been deemed possible with a work of such magnitude.

The large cities have all been canvassed by special agents, and through the adequate machinery of this firm the balance of the country has been quite thoroughly explored.

The "pages contain the names of nearly seventeen thousand persons practising dentistry in the States and Territories. Each name is accompanied by all the information regarding place and time of graduation or registration that the publishers have been able to obtain."

"At the commencement of the list for each State and Territory is a descriptive article embodying such matters as location, boundaries, extent in miles and acres, latitude and longitude, statistics regarding climate, temperature, population, . . . dental societies, and State Boards of Dental Examiners, and the full text of all laws relating to the profession."

That such a work should be free from errors is not to be expected. This is probably not possible of attainment with any directory, and especially so with one covering such an extended territory.

We notice, under the heading of Dental Societies of Pennsylvania, that there is much confusion in the use of the names "Odontographic" and "Odontological." These two are separate organizations, yet by a curious twisting of names the officers of the Odontological Society of Pennsylvania are placed under what should have been the Odontographic, with which they have no official connection. The attention of the publishers is also called to the omission of the "American Academy of Dental Science" under the same heading in Massachusetts. Other errors have been noted, but they are of minor character, and do not lessen to any extent the great value of this production as a work of reference.

The directory should be in the hands of every dentist in the country, and will be found indispensable to all colleges, boards of examiners, and business firms in connection with the practice of dentistry.

